# Neurobiological substrates of chronic low back pain (CLBP): a brain [^99m^Tc]Tc-ECD SPECT study

**DOI:** 10.1186/s41824-022-00145-2

**Published:** 2022-11-21

**Authors:** Erica Negrini Lia, Priscila Colavite Papassidero, Eduardo Barbosa Coelho, Fabíola Dach, Leonardo Alexandre-Santos, Ana Carolina Trevisan, Lucas Emmanuel Lopes e Santos, Jose Henrique Silvah, Vera Lúcia Lanchote, Oscar Della Pasqua, Lauro Wichert-Ana

**Affiliations:** 1 Department of Dentistry, School of Health Sciences, University of Brasilia (UnB), Brasilia, DF Brazil; 2 Department of Neurosciences and Behavioral Sciences, Ribeirão Preto Medical School, University of São Paulo (USP), Ribeirão Preto, SP Brazil; 3 Department of Internal Medicine, Ribeirão Preto Medical School, University of São Paulo (USP), Ribeirão Preto, SP Brazil; 4 Nuclear Medicine and PET/CT Laboratory, Department of Medical Imaging, Hematology and Clinical Oncology, Ribeirão Preto Medical School, University of São Paulo (USP), Ribeirão Preto, SP Brazil; 5 Department of Clinical Analysis, Food Science and Toxicology, School of Pharmaceutical Sciences of Ribeirão Preto, University of São Paulo, Ribeirão Preto, SP Brazil; 6 Clinical Pharmacology and Therapeutics, School of Life and Medical Sciences, University College London, London, UK; 7 Seção de Medicina Nuclear, Hospital das Clínicas – FMRP – USP, Av. Bandeirantes, 3900, CEP: 14048-900 Ribeirão Preto, SP Brasil

**Keywords:** Chronic low back pain, Brain SPECT, Neurobiological substrate, Numeric rating scale, Douleur Neuropathique 4 Questions

## Abstract

Recent neuroimaging studies have demonstrated pathological mechanisms related to cerebral neuroplasticity in chronic low back pain (CLBP). Few studies have compared cerebral changes between patients with and without pain in the absence of an experimentally induced stimulus. We investigated the neurobiological substrates associated with chronic low back pain using [^99m^Tc]Tc-ECD brain SPECT and correlated rCBF findings with the numeric rating scale (NRS) of pain and *douleur neuropathique en 4 questions* (DN4). Ten healthy control volunteers and fourteen patients with neuropathic CLBP due to lumbar disc herniation underwent cerebral SPECT scans. A quantitative comparison of rCBF findings between patients and controls was made using the Statistical Parametric Mapping (SPM), revealing clusters of voxels with a significant increase or decrease in rCBF. The intensity of CLBP was assessed by NRS and by DN4. RESULTS: The results demonstrated an rCBF increase in clusters A (occipital and posterior cingulate cortex) and B (right frontal) and a decrease in cluster C (superior parietal lobe and middle cingulate cortex). NRS scores were inversely and moderately correlated with the intensity of rCBF increase in cluster B, but not to rCBF changes in clusters A and C. DN4 scores did not correlate with rCBF changes in all three clusters. CONCLUSIONS: This study will be important for future therapeutic studies that aim to validate the association of rCBF findings with the pharmacokinetic and pharmacodynamic profiles of therapeutic challenges in pain.

## Introduction

Chronic low back pain (CLBP) is a common neurological disorder in the lumbosacral segment and persists over 12 weeks after its onset. CLBP affects about 10–20% of patients who do not present pain resolution (Deyo and Weinstein 2001). The etiology of CLBP is variable; discal hernia and lumbar spinal canal stenosis are the most common causes (Lee et al. 2020).

Early neuroimaging studies have demonstrated that pathophysiologic mechanisms of cerebral neuroplasticity are involved in CLBP (Nakamura et al. 2014), and related to structural and functional changes, being reversible after appropriate pain treatment (Seminowicz et al. 2011). CLBP patients presented several alterations, such as decreased gray substance in the prefrontal cortex, functional connectivity alterations of periaqueductal gray substance (PAG) (Yu et al. 2014), increased activation of the insula area, thalamus, amygdala, and the medial cingulate cortex (Rodriguez-Raecke et al. 2014).

Identifying specific regions involved in chronic pain and those related to each other, even if nonspecific, as well as the study of the development of pain and comorbidities associated with functional neuroimaging techniques, can allow the development of future target therapies (Tan et al. 2020; Isenburg et al. 2021).

However, few studies have compared cerebral changes between patients with and without pain in the absence of an experimentally induced stimulus (Schmidt-Wilcke 2015). Additionally, the minority of these studies have used the cerebral Single Photon Emission Computed Tomography (SPECT) to map the tridimensional regional cerebral blood flow (rCBF) (Bermo et al. 2021). Functional magnetic resonance (fMRI) is the most commonly used method (Yang et al. 2022).

This study aimed to investigate the neurobiological substrates associated with chronic low back pain using [^99m^Tc]Tc-ECD brain SPECT and correlate rCBF findings with the numeric rating scale (NRS) and *douleur neuropathique en 4 questions* (DN4).

## Methods

### Participants

Our institution’s Human Research Ethics Committee approved the present study, and all participants signed the informed consent. This case–control study compares the regional cerebral blood flow (rCBF) mapping between patients with CLBP and healthy volunteers. Participants were identified only by number, not name or initials. The study size was set based on previous similar studies. The study group consisted of 14 patients, aged 25–62 years old, with neuropathic CLBP due to lumbar disc herniation verified by structural magnetic resonance imaging (MRI), in follow-up at Neuropathic Pain Ambulatory from January to December 2016. The inclusion criteria were CLBP lasting longer than three months, with pain intensity equal to or greater than 3 in the numeric rating scale (NRS). The control group consisted of non-age matched healthy volunteers, aged 22 to 39 years old, and enrolled in the study after meeting the following criteria: 1) age above 18; 2) no complaints of acute or chronic pain at the time of evaluation; 3) absence of clinical, neurological, psychiatry, or cognitive disorders; and 4) no chronic use of medications. This study excluded patients with diabetes mellitus, arterial hypertension, anemia, severe cardiomyopathy, nephropathy, morbid obesity (BMI > 40), or any neurologic disease that presents neuropathic pain, besides chronic alcoholics and smokers. We also excluded participants with functional and anatomical alterations on brain SPECT. The study investigated the complete blood count, biochemical exams (total bilirubin and fractions, serum creatinine, glycemia, aspartate transaminase, gamma-glutamyl-transferase), and electrocardiogram of patients.

### Pain measurement

The Numerical Rating Scale (NRS) measured the intensity of CLBP from 0 (no pain) to 10 (worst pain). *Douleur Neuropathique en 4 Questions* (DN4), which combines seven items regarding symptoms and three findings on clinical examination, was also used for assessing CLBP.

### SPECT protocol

All patients underwent cerebral SPECT scans, and the tracer [^99m^Tc]Tc-ECD (ethyl cysteinate dimer) was injected at a maximum dose of 1,295 MBq (35 mCi). SPECT was performed during resting state, with eyes open, in a quiet and darkroom, refrained from talking and listening.

SPECT scans were acquired in a double-headed rotating gamma camera (SPECT/CT BrightView XCT, Philips Medical Systems Inc., Cleveland, OH, USA), equipped with a low-energy high-resolution collimator (LEHR), symmetrical acceptance energy window of 20%, and photopeak centered on 140 keV, using a 128 × 128 matrix, zoom factor of 1.0, and pixel size 2.13 mm. Data were collected in step-and-shoot mode over 360 degrees, in 128 projections (64 per head), with a total acquisition time of 30 min and about 100,000 counts/projection/head.

Tomographic images were processed in the workstation EBW (Extended Brilliance TM Workspace, Philips Medical Systems Inc., Cleveland, OH, USA), reconstructed in transaxial slices parallel to the orbitomeatal line, using Ordered Subset Expectation Maximization (OSEM) algorithm and a Butterworth filter order two and cut-off frequency 0.3. Chang’s method was applied over transaxial slices for the attenuation correction of photon effects (*µ* = 0.12 cm^−1^).

### SPM processing

The Statistical Parametric Mapping 8 (SPM8) software package (Wellcome Trust Center for Neuroimaging, University College of London, London, UK) converted brain SPECT images from DICOM to NIfTI format. The SPM software processed differences in rCBF between CLBP patients and healthy volunteers. The images were reoriented by setting the crosshairs to the anterior commissure, aligned and spatially normalized to the Montreal Neurological Institute (MNI) standard space using a 12-parameter affine transformation, followed by nonlinear transformations and trilinear interpolation. Normalized images were written with a bounding box equal to the image perfusion standard template, with 2 × 2 × 2 mm voxel dimensions. A binary mask was applied over the normalized images to remove all signals outside the brain structure and then convoluted with an isotropic kernel Gaussian function of 12 × 12 × 12 mm full-width at half maximum (FWHM) to smooth images before starting statistical analyses. Also, to remove the confounding effects of global brain counts between CLBP and healthy controls scans (two-sample t-test), the images were globally normalized for signal activity using proportional scaling with a threshold of 0.8 of the global mean. Thus, a voxel-by-voxel analysis compared each CLBP subject with the DN4 mean image (two-sample t-test). SPM-T maps were shown through a glass brain with threshold *p*-values = 0.001, uncorrected for multiple comparisons, at peak and cluster levels, with the cluster size being *k* = 125 voxels. Only clusters that overcame the correction for multiple comparisons with *p* < 0.05 were considered significant. The results displayed perfusion maps on the three-dimensional planes of a standard T1-MRI template.

### Statistical analysis

All statistical analyses and graphs were performed using Statistical Product and Service Solutions (SPSS) software (V24.0; IBM SPSS Statistics, IBM, Armonk, NY, USA). Spearman’s rank correlation coefficient (Spearman’s rho) is a nonparametric measure of rank correlation used to assess the relationship between NRS and DN4 scores. Kendall’s tau nonparametric rank correlation measured the association between signal intensity within clusters with increased or decreased rCBF on SPM and the NRS and DN4 scores and produced scatterplots. The statistical significance was set at *α* = 0.05.

## Results

### Participants

A total of 16 participants with CLBP participated in the study. Two patients were excluded by previous aneurysm clipping and meningioma. The final group consisted of 14 subjects (8 men and 6 women), with a mean age of 40.5 (± 9.4) years. The mean scores of NRS and DN4 were 5.7 (± 2.0) and 4.7 (± 2.6), respectively. Table 1 describes patients’ characteristics.

**Table 1:** Patients’ characteristics

Patient	Age(y)	Gender	LDH	Durationof pain	Medications in use
1 EMVB	38	F	L4–L5, Early fractured L2	1 y	Amitriptyline, sertraline, clonazepam
2 VC	44	M	L4–L5	2 y	–
3 LDC	31	M	L4–L5–S1	3 y	Acetaminophen
4 JAT	62	M	L4–L5	10 y	Cytidine, uridine, hydroxocobalamin
5 EAS	48	M	L2–L3–L4	6 y	–
6 ACAVS	33	F	L4–L5–S1	3 m	–
7 PHM	41	M	L4–L5	4 y	Anthraquinone, prednisone, thiamine
8 TCCM	35	F	L5–TV	1 y 8 m	–
9 IRR	48	F	L3–L4	3 y	Paroxetine, bupropion, clonazepam
10 IR	50	M	L4–L5	1y 6 m	Dexamethasone, betamethasone dipropionate, ketoprofen, celecoxibe, cyclobenzaprine
11 KDBBS	25	F	L4–L5	1 y	–
12 MECO	34	F	L4–L5	1 y 6 m	Nimesulide, dipyrone
13 CHFCF	37	F	L4–L5	12 y	–
14 JBC	41	F	L4–L5–TV	10 y	–

*y* years; *m* months; *M* Male; *F* Female; *LDH* location of the Lumbar Disc Herniation; *TV* Transitional Vertebra

**Fig. 1:**
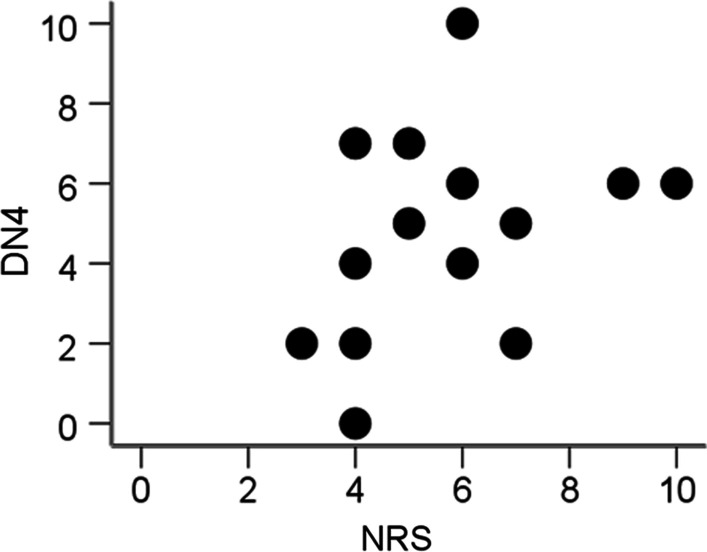
Scatter plot showing no correlation between DN4 and NRS scales for 14 patients (Spearman’s Kendall’s rho 0.357, *p* = 0.211). *DN4* Douleur Neuropathique en 4 Questions; *NRS* Numeric Rating Scale

The median NRS-observer and DN4 scores were 5.5 (IQR 4 to 7) and 5.0 (IQR 2 to 6.2), respectively. The NRS scores were 3 (*n* = 1, 7.1%), 4 (*n* = 4, 28.6%), 5 (*n* = 2, 14.3%), 6 (*n* = 3, 21.4%), 7 (*n* = 2, 14.3%), 9 (*n* = 1, 7.1%), and 10 (*n* = 1, 7.1%). The DN4 scores for the same group were 0 (*n* = 1, 7.1%), 2 (*n* = 3, 21.4%), 4 (*n* = 2, 14.3%), 5 (*n* = 2, 14.3%), 6 (*n* = 3, 21.4%), 7 (*n* = 2, 14.3%), and 10 (*n* = 1, 7.1%). The correlation between NRS and DN4 for patients with CLBP showed no significant association for all 14 patients (Spearman's rho 0.357, *p* = 0.211). (See Fig. 1).

### rCBF findings in patients with CLBP

**Fig. 2:**
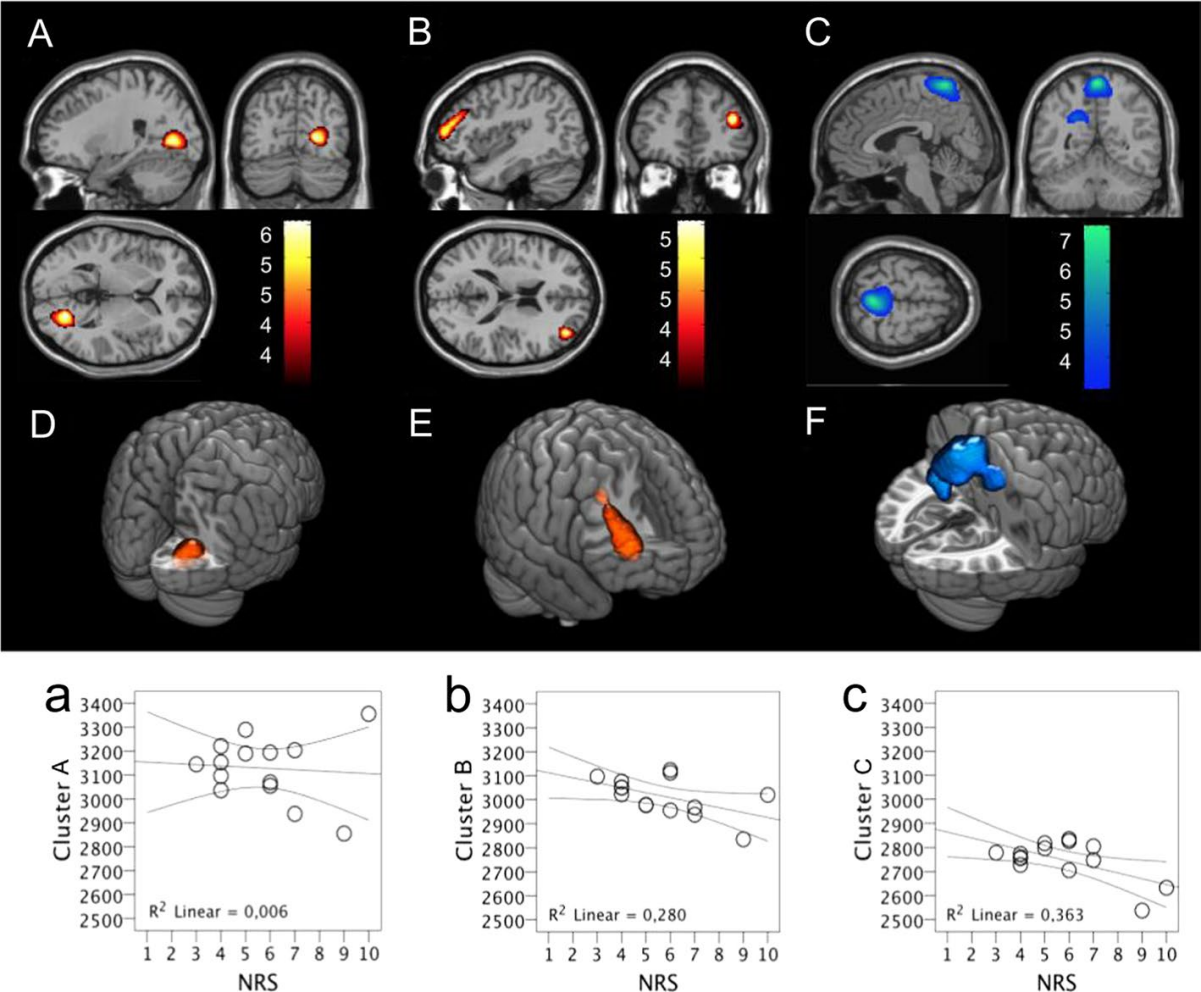
Results of the statistical parametric mapping (SPM) analysis showing brain regions with significant rCBF changes in 14 patients with CLBP, compared to ten healthy controls. The figure shows the overlay of clusters upon T1-weighted magnetic resonance imaging from the SPM template. Images show areas with a significant increase (**A**, **B**, **D**, and **E**) and decrease (**C** and **F**) of the regional cerebral blood flow (rCBF). The significant increase of rCBF was found in clusters **A** (3D view in **D**; R occipital lobe and R posterior cingulate gyrus) and **B** (3D view in **E**; R anterior prefrontal and dorsolateral frontal lobe). Cluster C presented a decrease of rCBF (3D view in **F**; bilateral parasagittal and postcentral parietal lobe and R middle cingulate cortex). Table 2 describes the Talairach coordinates. Results are shown in *p* value less than 0.05, corrected for multiple comparisons. A correlation coefficient with 95% CI according to Kendall’s tau non-parametric rank correlation was used to assess the relationship between the rCBF increase (**a**, **b**, **d**, and **e**) and decrease (**c** and **f**) and the results of the NRS (**a**–**c**) and DN4 (**d**–**f**) scores. The curved lines show the 95% CI around the regression line. In **b**, higher NRS scores were inversely and moderately correlated with the intensity of rCBF increase in cluster B (Kendall’s tau =  − 0.445, *p* = 0.033). In **a**, **c**, **d**, **e**, and **f**, there was no significant correlation between rCBF changes and NRS or DN4 scores. *NRS* Numeric Rating Scale; *DN4* Douleur Neuropathique en 4 Questions

Table 2 describes, and Fig. 2 shows the significant increase and decrease of rCBF changes in patients with CLBP. The right hemisphere presented the more significant increase of rCBF in occipital and posterior cingulate areas, frontal middle and inferior gyri, and the opercular frontal area. A cluster including bilateral regions of both parietal lobes and the right cingulate gyrus presented a significant decrease in rCBF.

**Table 2:** Brain regions of significant rCBF changes in CLBP patients

Brain regions	p*	Cluster volume (*k*)	Talairach coordinates x, y, z	Maximum voxel *Z* score
***Increased rCBF***				
*Cluster A—*R Occipital Lobe (Calcarine, Cuneus, Lingual, Middle Occipital Gyri), R Posterior Cingulate Gyrus	0.023	733	20, − 70, 4	4.35
*Cluster B—*R Frontal Lobe (Middle and Inferior Frontal Gyri), R anterior (BA 10), and dorsolateral (BA 46) prefrontal cortex	0.032	660	36, 44, 18	4.14
***Decreased *rCBF**				
Cluster* C—*L Parietal Lobe (Precuneus, Paracentral Lobe), R Parietal Lobe (Postcentral Gyrus, Paracentral Lobe) (BA 5 and 7) and R middle Cingulate Gyrus	0.000	2,774	0, − 52, 68	1.84

^*^p value of cluster significance; *R* right; *L* left; *BA* Brodmann Area

The correlation between individual rCBF changes in the three clusters and NRS and DN4 scores are shown in Fig. 2a–f. NRS scores were inversely and moderately correlated with the intensity of hyperperfusion in cluster B (Kendall's tau =  − 0.445, *p* = 0.033), but were not correlated to hyperperfusion in cluster A (Kendall's tau =  − 0.023, *p* = 0.911) or hypoperfusion in cluster C (Kendall's tau =  − 0.141, *p* = 0.502). DN4 scores were not correlated with any rCBF changes in clusters A (Kendall's tau = 0.000, *p* = 1.000), B (Kendall's tau =  − 0,185, *p* = 0.374), or C (Kendall's tau =  − 0.069, *p* = 0.739).

No patients presented abnormal findings on brain X-ray computed tomography (CT) (Table 3).

**Table 3:** Correlation between individual rCBF changes and NRS and DN4 pain scales

Patient	Cluster 1	Cluster 2	Cluster 3	NRS score	DN4 score
1	3193,57	2955,77	2835,19	6	10
2	2937,39	2968,07	2747,25	7	5
3	3189,93	2978,79	2796,52	5	7
4	3054,53	3124,16	2826,39	6	4
5	3203,44	2937,76	2804,74	7	2
6	3288,88	2976,96	2817,65	5	5
7	3144,52	3097,09	2778,34	3	2
8	3096,13	3050,18	2773,34	4	0
9	3152,95	3050,84	2757,04	4	2
10	3220,76	3023,11	2727,28	4	4
11	3355,50	3020,13	2632,55	10	6
12	3036,08	3075,75	2755,31	4	7
13	3069,31	3112,38	2705,10	6	6
14	2855,60	2835,26	2537,44	9	6
Mean (SD)	3128,47 132,95	3014,73 79,02	2749,58 81,22		

*NRS* Numeric Rating Scale; *DN4* Douleur Neuropathique en 4 Questions (Fishbain et al. 2014)

## Discussion

In this preliminary study, we investigated the neurobiological substrates associated with chronic low back pain due to lumbar disc herniation using [^99m^Tc]Tc-ECD brain SPECT in a small sample of 14 patients. The results revealed significant rCBF increase in the right prefrontal and dorsolateral frontal, occipital, and posterior cingulate areas, and rCBF decrease in the superior parietal lobe and middle cingulate cortex in patients with CLBP. These findings highlight the contribution of the perfusion SPECT in the identification of brain areas involved in chronic pain and may contribute to future therapeutic challenges in patients with CLBP, similar to previous studies in Fibromyalgia (Usui et al. 2010).

In our study, there was an rCBF increase in the right hemisphere, involving the prefrontal cortex, middle and inferior frontal gyri, and occipital and posterior cingulate areas. The prefrontal cortex (PFC) covers the anterior portion of the frontal lobe and deserves attention in pain processing. The medial prefrontal cortex (mPFC) is involved in two opposite roles, mediating antinociceptive effects and becoming chronic the pain (Ong, Stohler, and Herr 2018). fMRI studies with CLBP patients showed rest activation in the mPFC and cingulate cortex (DMN) (Kregel et al. 2015). The mPFC has been extensively studied in pharmacological and psychosocial pain management such as the use of antidepressants, acupuncture, cognitive behavioral therapy, meditation, music therapy, physical exercise, prayer, and others (Ong et al. 2018). Thus, our SPECT findings show that the PFC can be used as a therapeutic target for SPECT research in CLBP.

Another important target found in our study was the posterior cingulate cortex. This region is traditionally linked to visuospatial orientation (Vogt et al. 1996), episodic memory and pleasant stimuli (Maddock 1999), major depression and anxiety (Ho et al. 1996). Early study found an rCBF increase in the posterior cingulate cortex of patients with chronic neuropathic pain, suggesting its participation in the affective-motivational aspect of pain (Hsieh et al. 1995). Additionally, there may be a tendency to lateralization to the right hemisphere because of the affective processes involved in chronic neuropathic pain (Watanabe et al. 2018). Again, this region should also be evaluated in patients with CLBP.

A third target is the posterior cortex, that also constitute the “pain matrix”. The posterior insula, the adjacente suprasylvian operculum, and posterior parietal regions participate of this matrix, besides minor participation of the primary and secondary somatosensory cortices (S1, S2) (Garcia-Larrea and Bastuji 2018). Our study found more activation of the right occipital cortex in CLBP patients. Both the posterior parietal region and dorsolateral prefrontal cortex are involved in the cognitive-discriminative aspect of pain (Lindsay et al. 2021). Yet, we did not find SPECT changes in the cerebellum, contrary to previous studies that described increased rCBF in the bilateral posterior lobe of the cerebellum in patients with CLBP (Nakamura et al. 2014).

Conversely, we also found a decreased rCBF in a cluster including both superior parietal lobes and the middle cingulate gyrus. A recent study with osteopathic manipulative treatment found pos treatment rCBF decrease in the superior parietal lobe and, conversely, rCBF increase in the left posterior cingulate (Cerritelli et al. 2021). However, this was a postintervention study, different of our resting state investigation. It seems that superior parietal lobes somehow modulate the pain in CLBP.

Lastly, the numeric rating scale (NRS) of pain was inversely and moderately correlated with the intensity of rCBF increase in the right frontal lobe, and no correlation was observed between rCBF changes and *Douleur Neuropathique en 4 Questions* (DN4)*.* A study showed the good performance of DN4 in screening for various neuropathic pain syndromes. However, sensitivity varies by the syndrome. A positive DN4 was associated with greater pain catastrophizing, disability, and anxiety/depression, which may be explained by the severity of the disease (VanDenKerkhof et al. 2018). Our finding that the higher the numeric rating scale (a more subjective pain scale), the lower the activation of the frontal lobes, may suggest the involvement of frontal lobes more in the emotional part of pain management than directly to pain biology.

In summary, neuroimaging studies involving the evaluation of neuropathic pain show heterogeneous patterns of brain activation. Etiology of pain, lesion topography, symptoms, and stimulation procedures for activation neuroimaging studies are possible sources of heterogeneity (Watanabe et al. 2018; Moisset and Bouhassira 2007). The role of central pain amplification in patients with CLBP is undeniable (Gesieck et al., 2004). The history of pain, anatomical distribution, genetic constitution, and personality, may also alter the cerebral circuits involved in chronic pain processes and influence the interpretation of neuroimaging (Kupers and Kehlet 2006; Kupers and Kehlet 2006; Coninx and Stilwell 2021). At equal levels of pressure, patients with CLBP or fibromyalgia experienced more pain and showed more extensive neuronal activation in pain-related cortical areas than healthy controls. These findings are consistent with the occurrence of augmented central pain processing in patients with idiopathic CLBP (Giesecke et al. 2004).

Similarly to CLBP patients, patients with fibromyalgia present changes in perfusion SPECT (Kwiatek et al. 2000). Hypoperfusion was also found in the left culmen and a predominating hyperperfusion in the right precentral gyrus, posterior cingulate, superior occipital gyrus, cuneus, middle temporal gyrus, and left inferior parietal lobule, postcentral gyrus, and superior parietal lobule (Usui et al. 2010).

Particularly, and compared to responders to gabapentin, poor responders with fibromyalgia exhibited hyperperfusion in several brain and cerebellar regions, including frontal and cingulate cortices, with high positive likelihood ratios (Usui et al. 2010).

### Strengths and limitations

The present preliminary study contributes to the investigation of the neurobiological substrates of chronic lumbar back pain. Knowing the brain systems involved in CLBP and the functional activation and deactivation of these structures during the pain may represent the background for future pharmacokinetics and pharmacodynamic modeling studies. A limitation is the small sample of patients and healthy controls undergoing the study. However, the quantitative SPM technique offered sophisticated analysis tools that dispensed large samples, avoiding unnecessary exposure of patients to radiation. The group of participants with CLBP had a slightly older mean age than the control group.

## Data Availability

The data sets generated and/or analyzed during the current study are not publicly available due to patient confidentiality reasons but are available from the corresponding author on reasonable request and pending approval from the Ethics Committee of our University Clinical Hospital.
